# A Survey on Clustering Routing Protocols in Wireless Sensor Networks

**DOI:** 10.3390/s120811113

**Published:** 2012-08-09

**Authors:** Xuxun Liu

**Affiliations:** School of Electronic and Information Engineering, South China University of Technology, Guangzhou 510641, China; E-Mail: liuxuxun@scut.edu.cn; Tel./Fax: +86-20-8711-2490

**Keywords:** wireless sensor networks, clustering routing, cluster construction, data transmission, taxonomy

## Abstract

The past few years have witnessed increased interest in the potential use of wireless sensor networks (WSNs) in a wide range of applications and it has become a hot research area. Based on network structure, routing protocols in WSNs can be divided into two categories: flat routing and hierarchical or clustering routing. Owing to a variety of advantages, clustering is becoming an active branch of routing technology in WSNs. In this paper, we present a comprehensive and fine grained survey on clustering routing protocols proposed in the literature for WSNs. We outline the advantages and objectives of clustering for WSNs, and develop a novel taxonomy of WSN clustering routing methods based on complete and detailed clustering attributes. In particular, we systematically analyze a few prominent WSN clustering routing protocols and compare these different approaches according to our taxonomy and several significant metrics. Finally, we summarize and conclude the paper with some future directions.

## 1. Introduction

Owing to the advances and growth in Micro-Electro-Mechanical System (MEMS) technology and wireless communication technology, wireless sensor networks (WSNs) are becoming increasingly attractive for numerous application areas, such as military reconnaissance, disaster management, security surveillance, habitat monitoring, medical and health, industrial automation, *etc.* [1,2]. Thus, WSNs have managed to establish the connection between the physical world, the computing world and human society. In general, a WSN consists of a large number of tiny sensor nodes distributed over a large area with one or more powerful sinks or base stations (BSs) collecting information from these sensor nodes. All sensor nodes have limited power supply and have the capabilities of information sensing, data processing and wireless communication.

Routing is one of the critical technologies in WSNs. Opposed to traditional *ad hoc* networks, routing in WSNs is more challenging as a result of their inherent characteristics [3,4]. Firstly, resources are greatly constrained in terms of power supply, processing capability and transmission bandwidth. Secondly, it is difficult to design a global addressing scheme as Internet Protocol (IP). Furthermore, IP cannot be applied to WSNs, since address updating in a large-scale or dynamic WSN can result in heavy overhead. Thirdly, due to the limited resources, it is hard for routing to cope with unpredictable and frequent topology changes, especially in a mobile environment. Fourthly, data collection by many sensor nodes usually results in a high probability of data redundancy, which must be considered by routing protocols. Fifthly, most applications of WSNs require the only communication scheme of many-to-one, *i.e.*, from multiple sources to one particular sink, rather than multicast or peer to peer. Finally, in time-constrained applications of WSNs, data transmissions should be accomplished within a certain period of time. Thus, bounded latency for data transmissions must be taken into consideration in this kind of applications. Nevertheless, energy conservation is more important than quality of service (QoS) in most applications in that all sensor nodes are constrained with energy which is directly related to network lifetime.

Based on network structure, routing protocols in WSNs can be coarsely divided into two categories: flat routing and hierarchical routing. In a flat topology, all nodes perform the same tasks and have the same functionalities in the network. Data transmission is performed hop by hop usually using the form of flooding. The typical flat routings in WSNs include Flooding and Gossiping [5], Sensor Protocols for Information via Negotiation (SPIN) [6], Directed Diffusion (DD) [7], Rumor [8], Greedy Perimeter Stateless Routing (GPSR) [9], Trajectory Based Forwarding (TBF) [10], Energy-Aware Routing (EAR) [11], Gradient-Based Routing (GBR) [12], Sequential Assignment Routing (SAR) [13], *etc.* In small-scale networks flat routing protocols are relatively effective. However, it is relatively undesirable in large-scale networks because resources are limited, but all sensor nodes generate more data processing and bandwidth usage. On the other hand, in a hierarchical topology, nodes perform different tasks in WSNs and typically are organized into lots of clusters according to specific requirements or metrics. Generally, each cluster comprises a leader referred to as cluster head (CH) and other member nodes (MNs) or ordinary nodes (ONs), and the CHs can be organized into further hierarchical levels. In general, nodes with higher energy act as CH and perform the task of data processing and information transmission, while nodes with low energy act as MNs and perform the task of information sensing. The typical clustering routings protocols in WSNs include Low-energy Adaptive Clustering Hierarchy (LEACH) [14], Hybrid Energy-Efficient Distributed clustering (HEED) [15], Distributed Weight-based Energy-efficient Hierarchical Clustering protocol (DWEHC) [16], Position-based Aggregator Node Election protocol (PANEL) [17,18], Two-Level Hierarchy LEACH (TL-LEACH) [19], Unequal Clustering Size (UCS) model [20], Energy Efficient Clustering Scheme (EECS) [21,22], Energy-Efficient Uneven Clustering (EEUC) algorithm [23], Algorithm for Cluster Establishment (ACE) [24], Base-Station Controlled Dynamic Clustering Protocol (BCDCP) [25], Power-Efficient Gathering in Sensor Information Systems (PEGASIS) [26], Threshold sensitive Energy Efficient sensor Network protocol (TEEN) [27], The Adaptive Threshold sensitive Energy Efficient sensor Network protocol (APTEEN) [28], Two-Tier Data Dissemination (TTDD) [29], Concentric Clustering Scheme (CCS) [30], Hierarchical Geographic Multicast Routing (HGMR) [31], and *etc.* Clustering routing is becoming an active branch of routing technology in WSNs on account of a variety of advantages, such as more scalability, data aggregation/fusion, less load, less energy consumption, more robustness, *etc.*

In the last few years, a relatively large number of clustering routing protocols have been developed for WSNs. This paper is an attempt to comprehensively review and critically discuss the most prominent clustering routing algorithms that have been developed for WSNs. The goals of this survey can be summarized as follows: (1) To make a large audience aware of the existence and of the usually good performance of a number of clustering routing protocols in WSNs; (2) To facilitate the reading as well as to provide a sound framework by a detailed taxonomy of clustering routing algorithms; (3) To highlight a few strengths and weaknesses of the proposed algorithms with respect to the performance of the clustering routing methods in WSNs; (4) To help application designers identify alternative solutions and select appropriate strategies by comparison of different clustering routing approaches.

Recently, a few surveys of clustering routing methods for WSNs have been presented. These surveys mainly aim at outlining some characters of clustering and summarizing some popular clustering routing algorithms with comparison based on different attributes and performances. In this study, we present a comprehensive survey of different clustering routing protocols proposed in recent years. Our work differs from other surveys of WSN clustering routing algorithms as follows: (1) As far as we know, this survey is the first attempt to comprehensively review and critically discuss the most prominent clustering routing methods developed for WSNs; (2) It presents a novel taxonomy of clustering methods for WSNs, which is, to the best of our knowledge, the most comprehensive and fine-grained taxonomy of WSN clustering approaches at present; (3) This is the first time that the popular clustering routing protocols are surveyed based on the classification of different algorithm-stages; (4) It summarizes the previous surveys of WSN clustering routing protocols. As far as we know, our work is the first attempt to outline these contributions made by other researchers.

The remainder of this paper is organized as follows: Section 2 provides an overview of related work on clustering routing methods in WSNs. Section 3 outlines the advantages and objectives of clustering for WSNs. In Section 4, we present a novel taxonomy of clustering routing algorithms in WSNs. In Section 5, we systematically analyze the prominent WSN clustering routing protocols with discussion on their respective merits and demerits. In Section 6, we compare different approaches for WSN clustering routing. Finally, Section 7 summarizes and concludes this paper.

## 2. Related Work

A survey of clustering algorithms for WSNs was presented by Abbasi *et al.* [32]. The authors of that survey presented a taxonomy and classification of typical clustering schemes, then summarized different clustering algorithms for WSNs based on classification of variable convergence time protocols and constant convergence time algorithms, and highlighted their objectives, features, complexity, *etc.* Finally, these clustering approaches were compared based on a few metrics such as convergence rate, cluster stability, cluster overlapping, location-awareness and support for node mobility.

Arboleda *et al.* [33] presented a comparison survey between different clustering protocols. The authors of the survey discussed some basic concepts related to the clustering process, such as cluster structure, cluster types, clustering advantages, and briefly analyzed LEACH-based protocols as well as proactive and reactive algorithms in WSNs. The main characteristics of these protocols were compared and the evidences where they can be used currently were outlined.

Kumarawadu *et al.* [34] surveyed the clustering algorithms available for WSNs and classified them based on the cluster formation parameters and CH election criteria. The authors of the survey also studied the key design challenges and discussed the performance issues related clustering protocols based on the classification of identity-based clustering algorithms, neighborhood information based clustering algorithms, probabilistic clustering algorithms and biologically inspired clustering algorithms.

Different clustering schemes are discussed by Deosarkar *et al.* [35], with special emphasis on their CH selection strategies based on the classification of deterministic scheme, adaptive scheme and combined metric scheme. The costs of CH selection were compared with respect to cluster formation, distribution of CHs and creation of clusters. Besides, a need of more scalable, energy efficient and stable clustering scheme for data gathering in WSNs was put forward.

Jiang *et al.* [36] discussed a total of three prominent advantages of clustering methods for WSNs, such as more scalability, less overheads, and easy maintenance, and then present a classification of WSN clustering schemes based on a total of eight clustering attributes. The authors also analyzed altogether six popular WSN clustering algorithms, such as LEACH, PEGASIS, HEED, EEUC, and *etc.*, and compared these WSN clustering algorithms, including various attributes.

Maimour *et al.* [37] considered clustering routing protocols to achieve energy efficiency in WSNs and presented a review on clustering algorithms from the perspective of data routing. A simple classification of clustering routing protocols is proposed in the review. Totally nine typical clustering protocols including two classes, pre-established clustering routing algorithms and on-demand clustering routing algorithms, are summarized in respectively. Besides, some future research directions are presented in the review.

The operations of some clustering protocols were discussed in the survey presented in [38], and the advantages and limitations of each one of these algorithms were analyzed in brief. The authors of the survey selected only seven popular clustering algorithms for WSNs, such as LEACH, TL-LEACH, EECS, TEEN, APTEEN, and *etc.* Additionally, the survey compared these clustering protocols in terms of energy consumption and network lifetime.

A survey on clustering algorithms for WSNs was presented by Boyinbode *et al.* [39]. The main challenges for clustering algorithms were discussed and altogether nine popular clustering algorithms for WSNs such as LEACH, TL-LEACH, EECS, HEED, EEUC, *etc.* were simply summarized in the survey. The authors also compared these clustering algorithms based on metrics such as residual energy, uniformity of CH distribution, cluster size, delay, hop distance and cluster formation methodology.

A survey of state-of-the-art routing techniques for WSNs was presented in [40], whose authors outlined the clustering architecture in WSNs and presented a simple classification based on only three attributes, *i.e.*, parameters used for CH election, whether there exist a centralized control during clustering, and hops between nodes and CH in intra-cluster communication. Furthermore, the survey highlighted the challenges in clustering WSNs and briefly introduced a few clustering routing techniques.

Xu *et al.* [41] have made a simple survey of clustering routing protocols, including only six typical clustering algorithms. The authors of the survey simply compared these clustering routing algorithms based on some performance parameters, including energy conservation, network lifetime, data aggregation, robustness, scalability, security, and *etc.*

Another simple survey on clustering routing algorithms was given by Joshi [42]. Only eight popular clustering routing protocols are covered in this survey, such as LEACH, PEGASIS, TEEN, APTEEN, *etc.* The authors of the survey briefly compared these clustering routing approached based on energy conservation and the network lifetime.

An overview of Haneef and Deng [43] focuses on design challenges and comparative analysis of WSN clustering routing algorithms for improving the network lifetime. The authors of the overview analyzed many challenging factors that influenced design of routing protocols in WSNs, and presented a simple classification of routing protocols. Besides, many efficient clustering based classical WSN routing protocols with comparative analysis were discussed in the overview.

Finally, we summarize the previous work related to ours in Table 1, which highlights the main contributions of each author along with the year of the survey. However, our work differs from the others due to a few distinctive characteristics, which are described in last section.

## 3. Advantages and Objectives of Clustering

Compared with flat routing protocols in WSNs, clustering routing protocols have a variety of advantages, such as more scalability, less load, less energy consumption and more robustness. In this section, we summarize these advantages as well as the objectives of WSN clustering as follows:

### More Scalability

In s clustering routing scheme, sensor nodes are divided into a variety of clusters with different assignment levels. The CHs are responsible for data aggregation, information dissemination and network management, and the MNs for events sensing and information collecting in their surroundings. Clustering topology can localize the route set up within the cluster and thus reduce the size of the routing table stored at the individual sensor nodes [32,44]. Compared with a flat topology, this kind of network topology is easier to manage, and more scalable to respond to events in the environment [3].

### Data Aggregation/Fusion

Data aggregation/fusion, which is the process of aggregating the data from multiple nodes to eliminate redundant transmission and provide fused data to the BS, is an effectual technique for WSNs to save energy [45]. The most popular data aggregation/fusion method is clustering data aggregation, in which each CH aggregates the collected data and transmits the fused data to the BS [46]. Usually CHs are formed a tree structure to transmit aggregated data by multi-hopping through other CHs which results in significant energy savings [47].

### Less Load

Since sensors might generate significant redundant data, data aggregation or fusion has emerged as an important tenet and objective in WSNs. The main idea of data aggregation or fusion is to combine data from different sources to eliminate redundant data transmissions, and provide a rich and multi-dimensional view of the targets being monitored [3,4]. Many clustering routing schemes with data aggregation capabilities require careful selection for clustering approach. For clustering topology, all cluster members only send data to CHs, and data aggregation is performed at the CHs, which help to dramatically reduce transmission data and save energy. In addition, the routes are set up within the clusters which thus reduce the size of the routing table stored at the individual sensor nodes [32,44].

### Less Energy: Consumption

In clustering routing scheme, data aggregation helps to dramatically reduce transmission data and save energy. Moreover, clustering with intra-cluster and inter-cluster communications can reduce the number of sensor nodes performing the task of long distance communications, thus allowing less energy consumption for the entire network. In addition, only CHs perform the task of data transmission in clustering routing scheme, which can save a great deal of energy consumption.

### More Robustness

Clustering routing scheme makes it more convenient for network topology control and responding to network changes comprising node increasing, node mobility and unpredicted failures, *etc.* A clustering routing scheme only needs to cope with these changes within individual clusters, thus the entire network is more robust and more convenient for management. In order to share the CH responsibility, CHs are generally rotated among all the sensor nodes to avoid the single point of failure in clustering routing algorithms.

### Collision Avoidance

In the multi-hop flat model, the wireless medium is shared and managed by individual nodes, thus this model can result in low efficiency in the resource usage. On the other hand, in the multi-hop clustering model, a WSN is divided into clusters and data communications between sensor nodes comprise two modes, *i.e.*, intra-cluster and inter-cluster, respectively for data collection and for data transmissions. Accordingly, resources can be allocated orthogonally to each cluster to reduce collisions between clusters and be reused cluster by cluster [48]. As a result, the multi-hop clustering model is appropriate for large-scale WSNs.

### Latency Reduction

When a WSN is divided into clusters, only CHs perform the task of data transmissions out of the cluster. The mode of data transmissions only out of the cluster helps avoiding collisions between the nodes. Accordingly latency is reduced. Furthermore, data transmission is performed hop by hop usually using the form of flooding in flat routing scheme, but only CHs perform the task of data transmission in clustering routing scheme, which can decrease hops from data source to the BS, accordingly decrease latency.

### Load Balancing

Load balancing is an essential consideration aiming at prolonging the network lifetime in WSNs. Even distribution of sensor nodes among the clusters is usually considered for cluster construction where CHs perform the task of data processing and intra-cluster management. In general, constructing equal-sized clusters is adopted for prolonging the network lifetime since it prevents the premature energy exhaustion of CHs. Besides, multi-path routing is a method to achieve load balancing.

### Fault-Tolerance

Due to the applicability of WSNs in a good many dynamic scenarios, sensor nodes may suffer from energy depletion, transmission errors, hardware malfunction, malicious attacks and so on. With applications such as hurricane modeling and tracking envisioned to utilize a large number of small sensor nodes, the cost of each sensor node is constrained. Owing to significant constraints on the cost, and therefore on the quality of sensor motes, and the often hostile environments in which they are deployed, sensor networks are prone to failure. Thus, fault-tolerance is an important challenge in WSNs [49]. In order to avoid the loss of significant data from key sensor nodes, fault-tolerance of CHs is usually required in this kind of applications, thus effective fault-tolerant approaches must be designed in WSNs. Re-clustering is the most intuitive method to recover from a cluster failure, though it usually disarranges the on-going operation. Assignment of CH backup is a viable scheme for recovery from a CH failure.

### Guarantee of Connectivity

Sensor nodes usually transmit data to one or more BSs via a single-hop or multi-hop routing in WSNs, thus whether or not the data is successfully delivered to the BS is mainly determined by the connectivity of each node to its next hop node along the path. Furthermore, sensor nodes that cannot communicate with any other sensor node will get isolated and their data can never be transmitted to the BS. Therefore, guarantee of connectivity is an essential goal of clustering routing protocols in WSNs [3,4]. An important example is when some information concerning all the sensor nodes needs to be collected by a designated fusion node in clustering routing protocols [50].

### Energy Hole Avoidance

Generally, multi-hop routing is used to deliver the collected data to a sink or a BS. In those networks, the traffic transmitted by each node includes both self-generated and relayed traffic. Regardless of MAC protocols, the sensor nodes closer to the BS have to transmit more packets than those far away from the BS [51]. As a result, the nodes closer to the BS to deplete their energy first, leaving a hole near the BS, partitioning the whole network, and preventing the outside nodes from sending information to the BS, while many remaining nodes still have a plenty of energy. This phenomenon is called energy hole [52]. Mechanisms of energy hole avoidance, *i.e.*, energy consumption balancing, can be classified into three groups: node deployment, load balancing, as well as energy mapping and assigning [53]. Especially, uneven clustering is one of the methods of load balancing. In this method, a smaller cluster radius near the sink and a larger cluster radius away from the sink are defined respectively, so the energy consumption of processing data in inter-cluster is less for cluster with smaller radius, and thus more energy can be used to relay data from remote nodes [54]. On the other hand, it is not easy to analyze the optimization of cluster radius theoretically [55].

### Maximizing of the Network Lifetime

Network lifetime is an inevitable consideration in WSNs, because sensor nodes are constrained in power supply, processing capability and transmission bandwidth, especially for applications of harsh environments. Usually it is indispensable to minimize the energy consumption for intra-cluster communication by CHs which are richer in resources than ONs. Besides, sensor nodes that are close to most of the sensor nodes in the clusters should be prone to be CHs. Additionally, the aim of energy-aware idea is to select those routes that are expected to prolong the network lifetime in inter-cluster communications, and the routes composed of nodes with higher energy resources should be preferred.

### Quality of Service

The network applications and the functionalities of WSNs prompt the requirement of quality of service (QoS). Usually, effective sample, less delay and temporary precision are required. It is difficult for all the routing protocols to satisfy all the requirements of QoS, because some demands may breach one or more protocol principles. Existing clustering routing approaches in WSNs mainly focus on increasing energy efficient rather than QoS support. QoS metrics must be taken into account in many real-time applications, such as battle-target tracking, emergent-event monitoring, and *etc.*

## 4. Taxonomy of Clustering Schemes

In the literature, clustering attributes in WSNs, generally, can be roughly classified into cluster characteristics, cluster-head characteristics, clustering process and entire proceeding of the algorithm. In this section, we discuss a lot of detailed clustering attributes for WSNs, and propose a more comprehensive and fine-grained taxonomy compared to that of previous work. The categories included in the taxonomy are individually analyzed in the subsections that follow.

### 4.1. Classification of Clustering Attributes in WSNs

#### 4.1.1. Cluster Characteristics

##### Variability of Cluster Count

Based on variability of cluster count, clustering schemes can be classified into two types: fixed and variable ones. In the former scheme, the set of cluster-head are predetermined and the number of clusters is fixed. However, the number of clusters is variable in the latter scheme, in which CHs are selected, randomly or based on some rules, from the deployed sensor nodes.

##### Uniformity of Cluster Sizes

In the light of uniformity of cluster sizes, clustering routing protocols in WSNs can be classified into two classes: even and uneven ones, respectively with the same size clusters and different size clusters in the network. In general, clustering with different sizes clusters is used to achieve more uniform energy consumption and avoid energy hole.

##### Intra-Cluster Routing

According to the methods of inter-cluster routing, clustering routing manners in WSNs also include two classes: single-hop intra-cluster routing methods and multiple-hop ones. For the manner of intra-cluster single-hop, all MNs in the cluster transmit data to the corresponding CH directly. Instead, data relaying is used when MNs communicate with the corresponding CH in the cluster.

##### Inter-Cluster Routing

Based on the manners of inter-cluster routing, clustering routing protocols in WSNs include two classes: single-hop inter-cluster routing manners and multiple-hop ones. For the manner of inter-cluster single-hop, all CHs communicate with the BS directly. In contrast to it, data relaying is used by CHs in the routing scheme of inter-cluster multiple-hop.

#### 4.1.2. Cluster-Head Characteristics

##### Existence

Based on whether there exist cluster-heads within a cluster, clustering schemes can be grouped into cluster-head based and non-cluster-head based clustering. In the former schemes, there exist at least one CH within a cluster, but there aren't any CHs within a cluster in the latter schemes, such as some chain based clustering algorithms.

##### Difference of Capabilities

Based on uniformity of energy assignment for sensor nodes, clustering schemes in WSNs can be classified into homogeneous or heterogeneous ones. In homogeneous schemes, all the sensor nodes are assigned with equal energy, computation, and communication resources and CHs are designated according to a random way or other criteria. However, sensor nodes are assigned with unequal capabilities in heterogeneous environment, in which the roles of CHs are pre-assigned to sensor nodes with more capabilities.

##### Mobility

According to the mobility attributes of CHs, clustering approaches in WSNs also can be grouped into mobile and stationary manners. In the former manners, CHs are mobile and membership dynamically change, thus a cluster would need to be continuously maintained. Contrary to it, CHs are stationary and can keep a stable cluster, which is easier to be managed. Sometimes, a CH can travel for limited distances to reposition itself for better network performance [32].

##### Role

A CH can simply act as a relay for the traffic generated by the sensor nodes in its cluster or perform aggregation/fusion of collected information from sensor nodes in its cluster. Sometime, a cluster head acts as a sink/BS that takes actions based on the detected phenomena or targets [32]. It is worth mentioning, sometimes a CH acts in more than one role.

#### 4.1.3. Clustering Process

##### Control Manners

Based on control manners of clustering, clustering routing methods in WSNs can be grouped into centralized, distributed and hybrid ones. In centralized methods, a sink or CH requires global information of the network or the cluster to control the network or the cluster. In distributed approaches, a sensor node is able to become a CH or to join a formed cluster on its own initiative without global information of the network or the cluster. Hybrid schemes are composed of centralized and distributed approaches. In this environment, distributed approaches are used for coordination between CHs, and centralized manners are performed for CHs to build individual clusters.

##### Execution Nature

Considering the execution nature of cluster formation, clustering modes in WSNs can be classified into two classes: probabilistic or iterative ones. In probabilistic clustering, a probability assigned to all sensor nodes is used to determine the roles of the sensor nodes. In other words, each sensor node can independently decide on its own roles. Nevertheless, every node must wait until a certain number of iterations is achieved or for certain nodes to decide their roles before making a decision in iterative clustering manner.

##### Convergence Time

Considering the convergence time, clustering methods in WSNs can be grouped into variable and constant convergence time ones. The convergence time depends on the number of nodes in the network in variable convergence algorithms, which accommodate well to small-scale networks. After a fixed number of iterations, constant convergence time algorithms certainly converge regardless of the scale of the networks.

##### Parameters for CH Election

Based on the parameters used for CH election, clustering approaches can be categorized as deterministic, adaptive, and random ones. In deterministic schemes, special inherent attributes of the sensor nodes are considered, such as the identifier (ID), number of neighbors they have. In adaptive manners, CHs are elected from the deployed sensor nodes with higher weights, which includes such as residual energy, communication cost, and *etc.* In random modes, mainly used in secure clustering algorithms, CHs are elected randomly without regard to any other metrics like residual energy, communication cost, *etc.*

##### Proactivity

According to the proactivity of clustering routing, clustering routing methods can be grouped into proactive, reactive, and hybrid ones. In proactive networks, all routes between source and the BS are computed and maintained before they are really needed regardless of the data traffic. Once a message arrives, it travels through a predetermined route to the BS. In contrast, no predetermined routes exist in reactive networks, in which the routing is chosen when a message needs to be delivered from source to the BS. Hybrid approaches use a combination of the above two ideas. For this kind of clustering routing, sometimes proactive clustering mode is adopted, but at other times reactive mode is used. For instance, APTEEN [28] is a classical hybrid approach. According to the needs of users, this protocol adjusts some parameters and switches between proactive and reactive modes to transmit data.

##### Objectives

As discussed in the previous section, a few objectives have been pursued for cluster construction, such as data aggregation/fusion, load balancing, fault-tolerance, guarantee of connectivity, lifetime extension, quality of service, *etc.* Accordingly, clustering methods in WSNs can be classified into the above categories based on different objectives. It is worth mentioning that a clustering algorithm, generally, has more than one objective.

#### 4.1.4. Entire Proceeding of Algorithm

##### Algorithm Stages

In general, a complete clustering routing algorithm comprises two basic stages, *i.e.*, cluster construction and data transmission, but the consideration degree of algorithms may differ in different stages. Based on algorithm stages of whole process of clustering algorithms, clustering routing protocols in WSNs can be classified into cluster construction based and data transmission based ones. In the former algorithm, cluster construction is mainly discussed, while data transmission is concerned less or performed by a relatively simple way. As contrary to it, the latter one chiefly takes data transmission into account, but care less about cluster formation.

### 4.2. Taxonomy of Clustering Methods in WSNs

**Figure 1.**
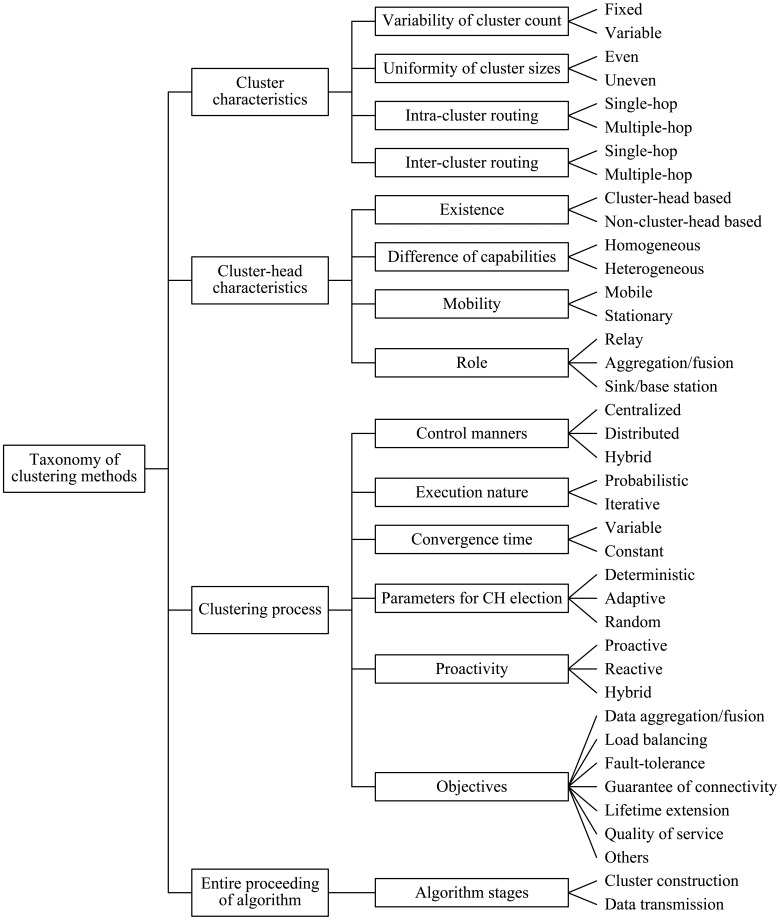
Taxonomy of Clustering Methods in WSNs.

In this subsection, we integrate the set of attributes that can be use to categorize and differentiate clustering methods for WSNs. Based on the discussion above, a relatively comprehensive and fine-grained taxonomy of clustering methods in WSNs is proposed, which is summarized in Figure 1.

## 5. Analysis of Prominent Clustering Routing Protocols in WSNs

In this section, we present a more comprehensive and critical survey of prominent clustering routing protocols for WSNs compared with previous work. We analyze 16 classical WSN clustering routing algorithms in detail based on the classification of different algorithm-stages, and highlight their characteristics with advantages and disadvantages.

### 5.1. Cluster-Construction Based Clustering Routing Protocols

#### 5.1.1. LEACH

Low-Energy Adaptive Clustering Hierarchy (LEACH), proposed by Heinzelman *et al.* [14], is one of the pioneering clustering routing approaches for WSNs. The basic idea of LEACH has been an inspiration for many subsequent clustering routing protocols. The main objective of LEACH is to select sensor nodes as CHs by rotation, so the high energy dissipation in communicating with the BS is spread to all sensor nodes in the network.

**Figure 2.**
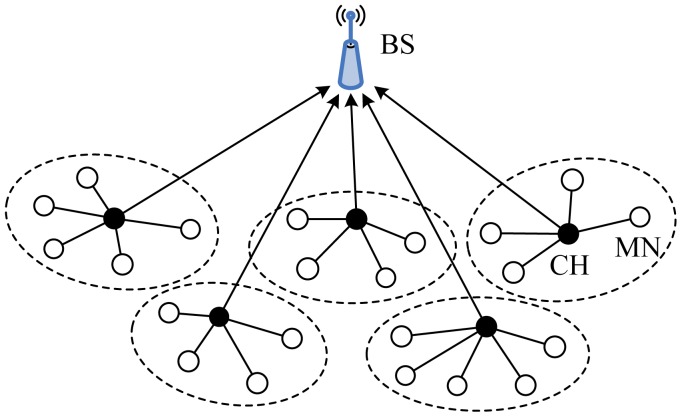
The Basic Topology of LEACH.

The operation of LEACH is broken up into lots of *rounds*, where each round is separated into two phases, the set-up phase and the steady-state phase. In the set-up phase the clusters are organized, while in the steady-state phase data is delivered to the BS. During the set-up phase, each node decides whether or not to become a CH for the current round. This decision is based on the suggested percentage of CHs for the network and the number of times the node has been a CH so far. This decision is made by the node choosing a random number between 0 and 1. The node becomes a CH for the current round if the number is less than the following threshold:

(1) $$T(n)=\left\{ \begin{array}{ll} \frac{P}{1-P\left(r\mathrm{mod}\frac{1}{P}\right)}, & \text{if}\;n\in G\\ 0, & \text{otherwise} \end{array}\right.$$

where *P* is the desired percentage of CHs, *r* is the current round, and *G* is the set of nodes that have not been elected CHs in the last 1/*P* rounds. When a node is elected CH successfully, it broadcasts an advertisement message to the other nodes. According to the received signal strength of the advertisement, other nodes decide to which cluster it will join for this round and send a membership message to its CH. In order to evenly distribute energy load among sensor nodes, CHs rotation is performed at each round by generating a new advertisement phase based on Equation (1). During the steady-state phase, the sensor nodes sense and transmit data to the CHs. The CHs compress data arriving from nodes that belong to the respective cluster, and send an aggregated or fused packet to the BS directly. Besides, LEACH uses a TDMA/code-division multiple access (CDMA) MAC to reduce inter-cluster and intra-cluster collisions. After a certain time, which is determined a priori, the network goes back into the set-up phase again and enters another round of CH election. Figure 2 showed the basic topology of LEACH.

LEACH is a completely distributed approach and requires no global information of network. In the literature, various modifications have been made to the LEACH protocol, which form LEACH family, such as TL-LEACH [19], E-LEACH [56], M-LEACH [57], LEACH-C [58], V-LEACH [59], LEACH-FL [60], W-LEACH [61], T-LEACH [62], *etc.* The advantages of LEACH include the following [63]: (1) Any node that served as a CH in certain round cannot be selected as the CH again, so each node can equally share the load imposed upon CHs to some ex0tent; (2) Utilizing a TDMA schedule prevents CHs from unnecessary collisions; (3) Cluster members can open or close communication interfaces in compliance with their allocated time slots to avoid excessive energy dissipation.

However, there exist a few disadvantages in LEACH as follows. (1) It performs the single-hop inter-cluster, directly from CHs to the BS, routing method, which is not applicable to large-region networks. It is not always a realistic assumption for single-hop inter-cluster routing with long communication range. Besides, long-range communications directly from CHs to the BS can breed too much energy consumption; (2) Despite the fact that CHs rotation is performed at each round to achieve load balancing, LEACH cannot ensure real load balancing in the case of sensor nodes with different amounts of initial energy, because CHs are elected in terms of probabilities without energy considerations. Sensor nodes, with lower initial energy, that act as CHs for the same number of rounds as other sensor nodes, with higher initial energy, will die prematurely. This could bring about energy holes and coverage problems; (3) Since CH election is performed in terms of probabilities, it is hard for the predetermined CHs to be uniformly distributed throughout the network. Thereby there exist the elected CHs that are concentrated in one part of the network and some nodes that have not any CHs in their vicinity; (4) The idea of dynamic clustering brings extra overhead. For instance, CH changes and advertisements may diminish the gain in energy consumption.

#### 5.1.2. HEED

Hybrid Energy-Efficient Distributed clustering (HEED) [15], introduced by Younis and Fahmy, is a multi-hop WSN clustering algorithm which brings an energy-efficient clustering routing with explicit consideration of energy. Different from LEACH in the manner of CH election, HEED does not select nodes as CHs randomly. The manner of cluster construction is performed based on the hybrid combination of two parameters. One parameter depends on the node's residual energy, and the other parameter is the intra-cluster communication cost. In HEED, elected CHs have relatively high average residual energy compared to MNs. Additionally, one of the main goals of HEED is to get an even-distributed CHs throughout the networks. Moreover, despite the phenomena that two nodes, within each other's communication range, become CHs together, but the probability of this phenomena is very small in HEED.

In HEED, CHs are periodically elected based on two important parameters: residual energy and intra-cluster communication cost of the candidate nodes. Initially, in HEED, a percentage of CHs among all nodes, *C*_prob_, is set to assume that an optimal percentage cannot be computed a priori. The probability that a node becomes a CH is:

(2) $$CH_{\text{prob}}=C_{\text{prob}}\frac{E_{\text{residual}}}{E_{\max}}$$

where *E*_residual_is the estimated current energy of the node, and *E*_max_ is a reference maximum energy, which is typically identical for all nodes in the network. The value of *CH*_prob_, however, is not allowed to fall below a certain threshold that is selected to be inversely proportional to *E*_max_. Afterwards, each node goes through several iterations until it finds the CH. If it hears from no CH, the node elects itself to be a CH and sends an announcement message to its neighbors. Each node doubles its *CH*_prob_ value and goes to the next iteration until its *CH*_prob_ reaches 1. Therefore, there are two types of status that a sensor node could announce to its neighbors: tentative status and final status. If its *CH*_prob_ is less than 1, the node becomes a tentative CH and can change its status to a regular node at a later iteration if it finds a lower cost CH. If it's *CH*_prob_ has reached 1, the node permanently becomes a CH. In HEED, every node elects the least communication cost CH in order to join it. On the other hand, CHs send the aggregated data to the BS in a multi-hop fashion rather than single-hop fashion of LEACH.

The advantages of the HEED protocol are as follows: (1) It is a fully distributed clustering method that benefits from the use of the two important parameters for CH election; (2) Low power levels of clusters promote an increase in spatial reuse while high power levels of clusters are required for inter-cluster communication. This provides uniform CH distribution across the network and load balancing; (3) Communications in a multi-hop fashion between CHs and the BS promote more energy conservation and scalability in contrast with the single-hop fashion, *i.e.*, long-range communications directly from CHs to the sink, in the LEACH protocol [64].

However, there are some limitations with HEED as follows: (1) The use of tentative CHs that do not become final CHs leave some uncovered nodes. As per HEED implementation, these nodes are forced to become a CH and these forced CHs may be in range of other CHs or may not have any member associated with them. As a result, more CHs are generated than the expected number and this also accounts for unbalanced energy consumption in the network [65]; (2) Similar to LEACH, the performing of clustering in each round imposes significant overhead in the network. This overhead causes noticeable energy dissipation which results in decreasing the network lifetime; (3) HEED suffers from a consequent overhead since it needs several iterations to form clusters. At each iteration, a lot of packets are broadcast. (4) Some CHs, especially near the sink, may die earlier because these CHs have more work load, and the hot spot will come into being in the network [66,67].

#### 5.1.3. DWEHC

Distributed Weight-based Energy-efficient Hierarchical Clustering protocol (DWEHC), proposed by Ding *et al.* [16], is a distributed clustering algorithm similar to HEED. The main objective of DWEHC is to improve HEED by building balanced cluster sizes and optimize the intra-cluster topology using location awareness of the nodes. Both DWEHC and HEED share some similarities including no assumptions about network size and density, and considering residual energy in the process of CH election. Every node implements DWEHC individually and the algorithm ends after several iterations that are implemented in a distributed manner.

**Figure 3.**
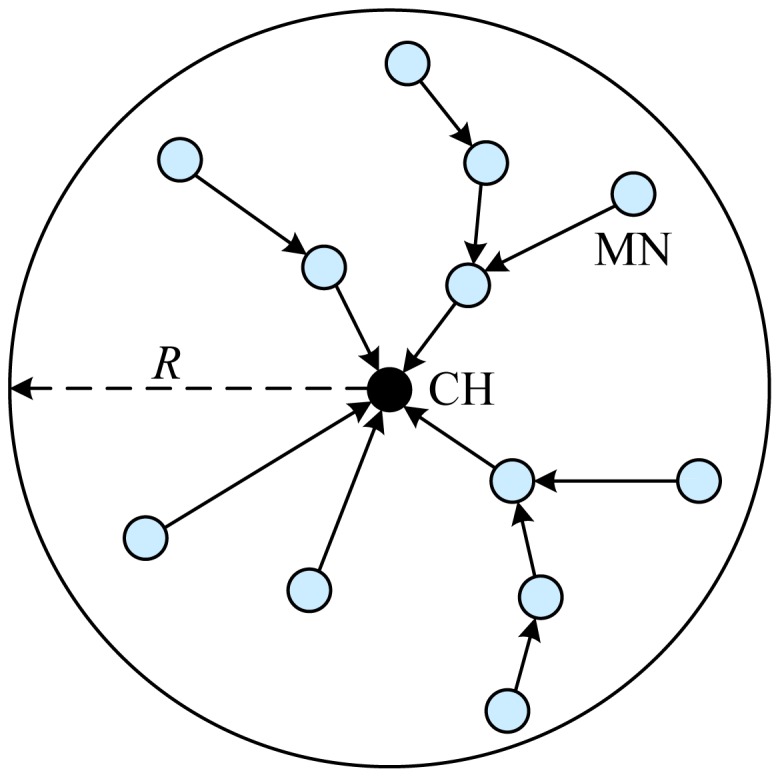
The Structure of Multi-level Cluster in DWEHC.

Different from LEACH and HEED, DWEHC creates a multi-level structure for intra-cluster communication and limits a parent node's number of children. Moreover, the only locally calculated parameter weight is defined for CH election in DWEHC. After locating the neighboring nodes in its area, each node calculates its weight according to:

(3) $$W_{\text{weight}}(s)=\frac{E_{\text{residual}}(s)}{E_{\text{initial}}(s)}\times \sum_{u}\frac{R-d}{6R}$$

where *E*_residual_(*s*) and *E*_initial_(*s*) are respectively residual and initial energy at node *s*, *R* is the cluster range that corresponds to how far from the CH to a node inside a cluster, and *d* is the distance between node *s* and the neighboring node *u*. In a neighborhood, according to Equation (3), the node with largest weight would be elected a CH and the other nodes become members. At this stage, MNs are considered as 1-level nodes and communicate directly with the CH. A MN can progressively adjust such membership in order to reach a CH using the least amount of energy. Given the node's knowledge of the distance to its neighbors, it can assess whether it is better to stay a 1-level member or become a *h*-level one where *h* is the number of hops from the CH to itself. If a MN can save energy while reaching its CH with more than one hop, it will become a *h*-level member. The process continues until all nodes achieve the most energy-efficient intra-cluster topology. Energy consumption for communicate in a cluster can be computed according to node's knowledge of the distance to its neighbors. To limit the number of levels, every cluster is assigned a cluster range *R* within which MNs should lay. The structure of multi-level cluster in DWEHC is illustrated in Figure 3. After running DWEHC, a node either becomes a CH or becomes a child in a cluster, and a node is covered by only one CH.

Intra-cluster communication is performed by TDMA. Each parent node polls its direct children and forwards the data to its parent node until the data reaches the CH. The parent node may aggregates several data packets from its children together with its own data into one packet. For inter-cluster communication, the CHs poll their first-level children, including their own data, and transmit to the BS.

The following is the advantages of DWEHC: (1) Like HEED, it is a fully distributed clustering method that is based on a function of the sensor's energy reserve and the proximity to the neighbors for CH election; (2) Considering energy reserves in CH election, DWEHC generates more well-balanced CHs distribution and achieves significantly lower energy consumption in intra-cluster and inter-cluster routing than HEED; (3) The clustering process of DWEHC terminates in a few iterations, and does not depend on network topology or size.

Some disadvantages of DWEHC are summarized as follows: (1) Similar to LEACH, single-hop inter-communication, directly from CHs to the BS, is performed in DWEHC. Thus DWEHC may result in significant amount of energy consumption, and is not applicable to large-region networks; (2) In the process of cluster formation, the iterative nature in both DWEHC and HEED produces a relatively high control message overhead compared to other protocols.

#### 5.1.4. PANEL

Position-based Aggregator Node Election protocol (PANEL) [17,18], presented by Buttyan and Schaffer, is a position-based clustering routing protocol for WSNs. With respect to other CH election protocols, PANEL supports asynchronous sensor network applications where the sensor node readings are fetched by the BSs. The main goal of PANEL is to elect aggregators, *i.e.*, CHs, for reliable and persistent data storage applications.

**Figure 4.**
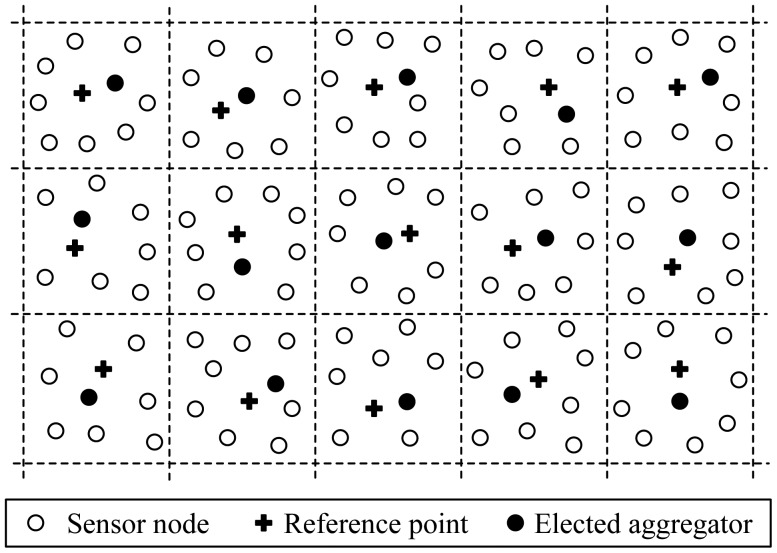
Illustration of the Geographical Clustering in PANEL.

PANEL assumes that the nodes are deployed in a bounded area, which is partitioned into geographical clusters. The clustering is determined before the deployment of the network, and each node is pre-loaded with the geographical information of the cluster to which it belongs. PANEL introduces a notion of reference point. At the beginning of each epoch, a reference point *R_j_* is computed in each cluster *j* by the nodes in a distributed manner in terms of the epoch number, as follows:

(4) $${\vec{R}}_{j}={\vec{Q}}_{j}+\vec{Q}$$

where *Q⃗j* is the position of the lower-left corner of cluster *j*. Furthermore, the current epoch number *e* is known by every node and the computation consists in calling a pseudo-random function *H*(e) that maps *e* to a relative position *Q⃗* nside the cluster, *i.e.*,:

(5) $$H(e)=\vec{Q}$$

where *Q⃗* ∈ (−*δd*, *d* + *δd*) × (−*δd*, *d* + *δd*), *d* is the size of the cluster, and *δ* < 1 is a parameter which expresses the magnitude of this re-sizing operation in percent of the original cluster size *d*. Once the reference point is computed, the node that is the closest to the reference point will be elected the CH for the given epoch. The reference points of the clusters will be re-computed and the CH election procedure will be re-executed in next epochs. This CH election procedure ensures load balancing in PANEL because each node of the cluster can become CH with almost the same probability. The illustration of the geographical clustering in PANEL is shown in Figure 4.

The CH election procedure needs intra-cluster communications. PANEL takes advantage of these communications to establish routing tables for intra-cluster routing. Especially, at the end of the CH election procedure, the nodes also are conscious of the next hop towards the CH elected for the current epoch. Moreover, a position-based routing protocol is introduced in PANEL. The intra-cluster routing is used to route a message to the aggregator of a given cluster if that messages is already inside the cluster. The intra-cluster routing of PANEL takes advantage of the fact that the nodes within the cluster communicate during the aggregator election procedure.

The following are the main merits of PANEL: (1) This protocol is an energy-efficient protocol that ensures load balancing because each node is elected aggregator, *i.e.*, CH, nearly equally frequently. Besides, data aggregation is performed and communication load is reduced, accordingly PANEL can prolong the network lifetime; (2) The outstanding feature of PANEL that makes it different from other data-aggregation based clustering protocols is that besides synchronous scenes, it also supports asynchronous applications.

The main limitations of PANEL are discussed as follows: (1) The assumption that the clusters are determined before deployment and thus cannot be applied to WSN dynamics; (2) Geographical position information of the nodes is used to determine which node should be the aggregators. This is a restriction in WSNs, because the geographical position is not always available without special condition, such as GPS-like hardware and software; (3) A crucial assumption of PANEL, described by the authors of PANEL, is that the nodes within a cluster form a connected sub-network. If this assumption is not satisfied, and the sub-network within a cluster is partitioned, then some nodes will not hear the announcement of the node closest to the reference point, and they will elect another node as aggregator.

#### 5.1.5. TL-LEACH

Two-Level Hierarchy LEACH (TL-LEACH), introduced by Loscrì *et al.* [19], is an extension to the algorithm of LEACH. TL-LEACH uses the following two techniques to achieve energy and latency efficiency: randomized, adaptive, self-configuring cluster formation and localized control for data transfers. In TL-LEACH, a CH collects data from MNs as original LEACH, but instead of transmitting data to the BS directly, it uses a part of CHs that lies between the CH and the BS as a relay station.

**Figure 5.**
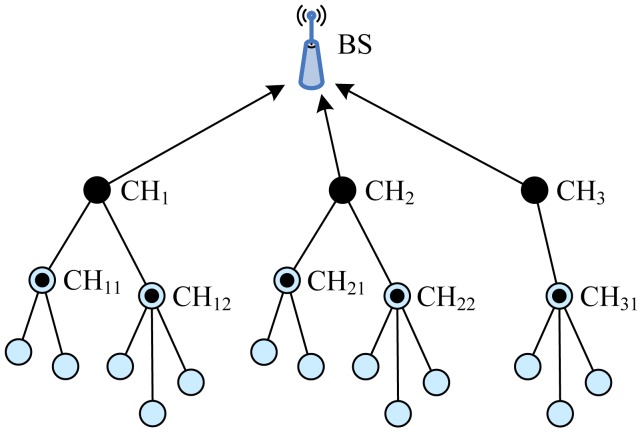
The Two-level Hierarchy in TL-LEACH.

TL-LEACH introduced two-level hierarchy as shown in Figure 5: top CHs called primary cluster heads (CH*_i_*), second level represented from secondary cluster heads (CH*_ij_*) and ONs. The algorithm is composed from four basic phases: advertisement phase, cluster setup phase, schedule creation and data transmission. In the first phase, each node decides whether it become a primary CH, secondary CH or ON in each round which is the same as that of LEACH. If a node is elected a primary CH, it must advertise other nodes. The mechanism used in this phase is carrier sense multiple access (CSMA). Thereafter, secondary CH nodes send the advertisement to the ONs. In this phase, each secondary CH decides to which primary CH it belongs and sends an advertisement message to its primary CH. In the same way, each ON must decide which secondary CH it belongs to and informs it through an opposite message. In the third phase, each primary CH creates a TDMA schedule assigning each node in its group a slot to transmit. Each primary CH chooses a CDMA code and informs all the nodes at second level in its group to use this code. In the same way, each secondary CH can transmit this information to ONs in its group using both the code and the schedule from the primary CH. In the last phase, clusters are created and each node can transmit in respect to the TDMA schedule decided by its primary CH.

The advantages of TL-LEACH are as follows: (1) TL-LEACH uses random rotation of local cluster BSs, *i.e.*, primary CHs and secondary CHs, which can bring about better energy load distribution across the network; (2) TL-LEACH uses localized coordination, which is conductive to scalability and robustness in the network; (3) Compared with LEACH, the scheme of two-levels clustering leads to less average transmission distance, and less nodes are required to transmit far distances to the BS via TL-LEACH. This effectively reduces the total energy consumption.

However, there exist a few disadvantages of TL-LEACH as follows: (1) Despite that the average transmission distance is decreased in comparison with LEACH, the two-hop inter-cluster routing of TL-LEACH is still not applicable to large-range networks, because it uses only two hops for data transmission from sources to the BS, and long-distance communications can breed much energy consumption; (2) CH election without energy considerations assumes an ideal homogeneous network and can not ensure real load-balancing in case of nodes with different amount of initial energy.

#### 5.1.6. UCS

Unequal Clustering Size (UCS) model [20] was proposed by Soro and Heinzelman for network organization in order to balance energy consumption of CHs, thus increasing the network lifetime. UCS is the first unequal clustering model for WSN organization. It is assumed that the positions of the CHs are determined *a priori*, with all CHs arranged symmetrically in concentric circles around the BS which is located in the center of the network, thus it's easy to control the actual sizes of different clusters.

**Figure 6.**
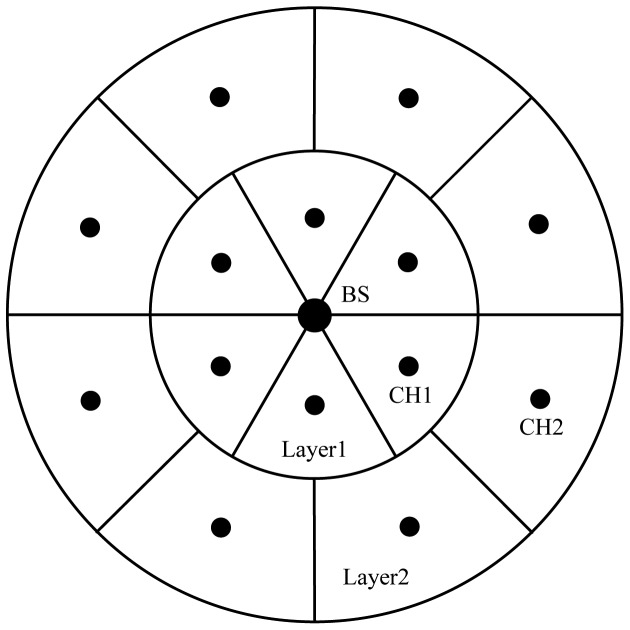
Pie Shaped Clusters Arranged in Two Layers in UCS.

In UCS, the sensing field is assumed to be circular and is divided into two concentric circles, called layers. In order to simplify the theoretical analysis, the authors approximate the sensing field as pie shaped field with a multiple-layer network, shown in Figure 6. It is assumed that all clusters in one layer have the same size and shape, but the sizes and shapes of clusters in the two layers are different. The position of a CH within the cluster boundaries determines the overall energy consumption of nodes that belong to the cluster. To keep the total energy dissipation within the cluster as small as possible, every CH should be positioned at the center of the cluster. CHs are deterministically deployed in the network and are assumed to be super nodes which are much more expensive than MNs. By varying the radius of the first layer around the BS, while assuming a constant number of clusters in every layer, the area covered by clusters in each layer can be changed, and accordingly the number of nodes contained in a particular cluster can be changed. Data transmission is done through multiple hops, where every CH chooses to forward its data to the closest CH in the direction of the BS.

The advantages of UCS are discussed as follows: (1) By changing the number of nodes in every cluster with respect to the expected communication load, UCS can maintain more uniform energy consumption among the CHs. Therefore, the total energy dissipated for every CH is similar and UCS can prolong network lifetime compared with the model of Equal Clustering Size (ECS); (2) Using the two-layered network model and two-hop inter-cluster communication method, UCS results in a shorter average transmission distance compared with LEACH, thus effectively reduces the total energy consumption.

However, there exist a few limitations in UCS as follows: (1) UCS is constrained by the assumption that the network is heterogeneous, and CHs are performed by super nodes all the time and are deployed at pre-determined locations. That is to say, it lacks universality [68]; (2) CHs are required to locate in the center of the cluster, thus a key factor, residual energy of nodes, is not considered in UCS; (3) Similar to TL-LEACH, despite that the average transmission distance is decreased in comparison with LEACH, the two-hop inter-cluster routing of UCS is still not applicable to large-range networks, because it uses only two hops for data transmission from sources to the BS, and long-distance communications need much energy consumption.

#### 5.1.7. EECS

Energy Efficient Clustering Scheme (EECS), proposed by Ye *et al.* [21,22], is a clustering algorithm which better suits the periodical data gathering applications. EECS is a LEACH-like scheme, where the network is partitioned into several clusters and single-hop communication between the CH and the BS is performed. In EECS, CH candidates compete for the ability to elevate to CH for a given round. This competition involves candidates broadcasting their residual energy to neighboring candidates. If a given node does not find a node with more residual energy, it becomes a CH. Different from LEACH for cluster formation, EECS extends LEACH by dynamic sizing of clusters based on cluster distance from the BS.

In EECS, a node chooses the CH by considering not only saving its own energy but also balancing the workload of CHs, *i.e.*, two distance factors: *d*(*P_j_*, *CH_i_*) and *d*(*CH_i_*, *BS*). A weighted function *cost*(*j,i*) is introduced in EECS for the ordinary node *P_j_* to make a decision, which is:

(6) $$\text{cost}(j,i)=((1-w(P_{j}))w\times f(P_{j},CH_{i})+w(P_{j})\times g(CH_{i})$$

and node *P_j_*, chooses cluster head *CH_i_* with the minimal {*cost*}to join. In Equation (6)*f* and *g* are two normalized functions for the distance *d*(*P_j_*, *CH_i_*) and *d*(*CH_i_*, *BS*), respectively:

(7) $$f(P_{j},CH_{i})=\frac{d(P_{j},CH_{i})}{d_{f\_\text{max}}}$$

(8) $$g(CH_{i})=\frac{d(CH_{i},BS)-d_{g\_\text{min}}}{d_{g\_\text{max}}-d_{g\_\text{min}}}$$

where *d_f_max_* = exp(max{*d*(*Pj*, *CH_i_*)}), *d_g_max_* = max{*d*(*CH_i_*, *BS*)} and *d_g_min_* = min{*d*(*CH_i_*, *BS*)}. *w* is the function of *P_j_* as follows:

(9) $$w(P_{j})=c+(1-c)\sqrt{\frac{d(P_{j},BS)}{\left(d_{g\_\text{max}}-d_{g\_\text{min}}\right)}}$$

Function *f* in *cost* guarantees that nodes choose the closest CH in order to minimize the intra-cluster communication cost, while function *g* makes the nodes join the CH with small *d*(*CH_i_*, *BS*)} to alleviate the workload of the CHs farther from the BS. Function *w* is the weighted factor for the tradeoff between *f* and *g*. Furthermore, the optimal value of weighted factor *c* in the function *w* depends on the specific network scale.

The advantages of EECS are summarized as follows: (1) Based on energy and distance, EECS constructs balancing point between intra-cluster energy consumption and inter-cluster communication load; (2) Clustering is performed by dynamic sizing based on cluster distance from the BS. This addresses the problem that clusters with a larger distance to the BS require more energy for transmission than those with a shorter distance, and bring about low message overheads and uniform distribution of CHs compared to LEACH.

However, there exist a few advantages in EECS as follows: (1) Account of single-hop communications in EECS, long-range transmissions directly from CHs to the BS can lead to much energy consumption. Hence it is not suitable for large-range networks; (2) EECS requires more global knowledge about the distances between the CHs and the BS, and the task of global data aggregation adds overheads to all sensor nodes; (3) EECS produces much more control overhead complexity because all nodes must compete for becoming CHs.

#### 5.1.8. EEUC

Energy-Efficient Uneven Clustering (EEUC) algorithm, proposed by Li *et al.* [23], is a clustering and distributed competitive algorithm, where CHs are elected by localized competition, which is unlike LEACH. Every node has a pre-assigned competitive range, which is smaller as it gets close to the BS. This makes EEUC an unequal clustering approach for the purpose of balancing energy consumption among CHs and solving the hot spots problem. During the process of CH election in EEUC, each node generates a random number, and only the node whose number is greater than a threshold will be activated for CH election by broadcasting compete message within a competition radius which is determined by its distance to the BS. The competition radius of node *s_i_* is given by:

(10) $$s_{i}.R_{\text{comp}}=\left[1-c\frac{d_{\text{max}}-d(s_{i},BS)}{d_{\text{max}}-d_{\text{min}}}\right]R_{\text{comp}}^{0}$$

where *R*0 *comp* is the maximum competition radius which is predefined, *d_max_* and *d_min_* denote the maximum and minimum distance between sensor nodes and the BS, *d*(*s_i_*, *BS*) is the distance between node *s_i_* and the BS, *c* is a constant coefficient between 0 and 1. According to Equation (10), the node's competition range decreases as its distance to the BS decreasing. Accordingly clusters closer to the BS have smaller cluster sizes, thus they will consume lower energy during the intra-cluster data processing, and can preserve more energy for the inter-cluster relay traffic. If a sensor node decides to participate to the competition, which is based on the residual energy of each tentative CH, it becomes a tentative CH. Then, tentative CHs in local regions compete in order to become a real CH.

In EEUC, multi-hop routing is used for inter-cluster communication. The CHs choose relay nodes for data transmission according to the nodes' residual energy and distance to the BS. In other words, a CH would choose the one with more residual energy as its relay node from the two whose communication cost are the least among all of its neighbor CHs.

According to above discussion, the disadvantages of EEUC are as follows: (1) To address the hot spots problem, EEUC introduces an unequal clustering mechanism to balance the energy consumption among CHs. Accordingly, the unequal clustering mechanism in EEUC improves the network lifetime over LEACH and HEED; (2) Based on communication cost, this protocol can save more energy via inter-cluster multi-hop routing mechanism in steady state phase, because a CH would choose its relay node from the two whose communication cost are the least among all of its neighbor CHs.

However, there are several drawbacks in EEUC as follows: (1) Performing of clustering in each round imposes significant overhead, because each node must broadcast and receive a large amount of competition message for CH election, even though most of them cannot win and most of the elected nodes are not suitable to be as CHs; (2) The extra global data aggregation can result in much overhead for all nodes and deteriorate the network performance; (3) The routing scheme can result in new hot spots, in that only one of the two nodes whose communication costs are the least among the neighbor CHs can be relay nodes, even though both of them have little residual energy.

#### 5.1.9. ACE

Algorithm for Cluster Establishment (ACE) [24], presented by Chan and Perrig, employs an emergent algorithm, which is any computation that achieves formally or stochastically predictable global effects, by communicating directly with only a bounded number of immediate neighbors and without the use of central control or global visibility. One of the main distinguishing characteristics of emergent protocols over other localized protocols is the existence of feedback during protocol operation.

The main idea of ACE is to allow a node to assess its potential as a CH before becoming one and retire if it is not the best CH at the moment. The algorithm works in iterations that do not have to be synchronized at the individual nodes. ACE has two logical parts: the spawning of new clusters and the migration of existing clusters. When a node decides to become a CH, it spawns of new cluster by broadcasting an invitation message to recruit its neighbors. Upon getting the invitation, a neighboring node joins the new cluster and becomes a follower of the new cluster. At any moment, a node can be a follower of multiple clusters while the protocol is running. However, the node can be a loyal follower, *i.e.*, a member which belongs to only one cluster. Migration is a process in which the best candidate for being CH is elected again. Each CH will periodically check the ability of its followers to determine which is the best candidate for the new leader of the cluster. The CH will retire if one of these followers has more followers than it does. The node, considered as the best candidate for CH, would have the largest number of followers while minimizing the amount of overlap with existing clusters. Once the best candidate is determined by the current CH, it will assign the best candidate as the new CH and abdicate its position as the old CH. Thus, the position of the cluster will appear to migrate in the direction of the new CH. Accordingly, some of the former followers of the old CH do not belong to the new cluster, while some new nodes near the new CH become new followers of the new cluster.

The characteristics and advantages of ACE are summarized as follows: (1) ACE is an emergent algorithm that uses feedback to induce the formation of a highly efficient cover of uniform clusters over the network; (2) Minimizing the number of CHs would not only generate efficient cover of the whole network but also minimizes the cluster overlaps. This also improves the efficiency of the algorithms that executes at the level of the CHs; (3) ACE is very robust because it can easily repair structure damage caused by node failure and can also integrate new nodes in the network.

Nevertheless, there are several limitations in ACE as follows: (1) The important factor, energy, is not considered during the process of CH election. That is to say, those nodes with low residual energy may be elected CHs and work until death; (2) It is hard to decide the number of iterations for cluster formation while satisfying the communication cost requirements and energy consumptions; (3) It is obvious that migratory mechanism needs a large amount of information exchange among sensor nodes, thus this protocol adds additional overheads.

#### 5.1.10. BCDCP

Base-Station Controlled Dynamic Clustering Protocol (BCDCP), introduced by Muruganathan *et al.* [25], is a centralized clustering routing protocol with the BS being capable of complex computation. The main idea of BCDCP is the cluster formation where each CH serves an almost equal number of MNs to balance CH overload and uniform CH placement throughout the network.

At the beginning of cluster setup, the BS receives information on the residual energy from all the nodes in the network. Based on this information, the BS first computes the average energy level of all the nodes in the network, and then chooses a set of nodes whose energy levels are above the average value. Only the nodes from the chosen set, *i.e.*, those with sufficient energy, can be elected CHs for the current round, while those with low energy can prolong their lifetime by performing the task of ONs. Based on the chosen set, the BS computes the number of clusters and performs the task of clustering, which is accomplished in terms of an iterative cluster splitting algorithm. This algorithm first splits the network into two sub-clusters, and proceeds further by splitting the sub-clusters into smaller clusters. This process will be repeated until the desired number of clusters is achieved. At each iteration of cluster splitting, two nodes that have the maximum separation distance are chosen for CHs from the chosen set where all the nodes are eligible to become CHs. Then, each of the remaining nodes in the current cluster is grouped with one CH or the other, whichever is closest. After balancing the two groups which have approximately the same number of nodes, the two sub-clusters are formed.

**Figure 7.**
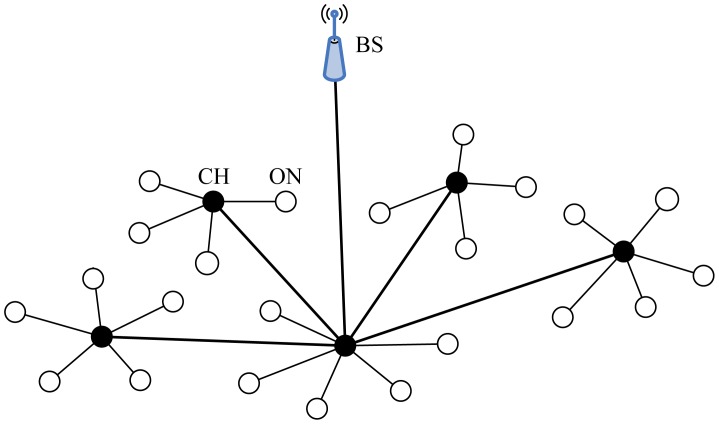
The Topology of the Network in BCDCP.

In BCDCP, a multi-hop routing scheme is adopted to transfer the sensed data to the BS. Once the clusters and the CHs have been identified, the BS chooses the lowest-energy routing path and transfer information to the nodes along with the details on cluster groupings and selected CHs. The routing paths are selected by first connecting all the CHs by means of the Minimum Spanning Tree (MST) approach [69], which minimizes the energy consumption for each CH, and then randomly choosing one CH to forward the data to the BS. By randomizing the CH transmissions to the BS, the transmission burden is distributed almost evenly among all CHs in BCDCP. Figure 7 is the topology of the network in BCDCP.

BCDCP utilizes a high-energy BS to set up clusters and uses MST [69] to connect CHs and randomly chooses a leader to send data to the BS. The advantages of BCDCP include the following [63]: (1) Clusters and transmission paths are constructed by the BS, thus BCDCP resolves the problem of CH distribution and ensures similar power dissipation of CHs; (2) TDMA is employed to schedule the time slots of cluster members; this allows sensor nodes to open communication interfaces only if data transmissions are required, which means energy can be saved at the same time.

However, there exist a few disadvantages in BCDCP as follows: (1) BCDCP is a centralized algorithm which brings worse scalability and robust to large networks than distributed algorithms; (2) Each node needs to transmit information regarding its location and energy level to the BS during the process of cluster formation. Accordingly it increases the design complexity and the energy consumption of the nodes to some ex0tent; (3) Due to the single-hop routing scheme, it is not appropriate for long-distance communications, which result in much energy consumption. Therefore, BCDCP is not adaptive to applications in large-range networks; (4) BCDCP is not suitable for reactive networks where the user is not interested in periodic data retrieval, while the nodes only need to respond to events of certain significance in reactive networks.

### 5.2. Data-Transmission Based Clustering Routing Protocols

#### 5.2.1. PEGASIS

Power-Efficient Gathering in Sensor Information Systems (PEGASIS), proposed by Lindsey *et al.* [26], is an improvement of LEACH. The main idea of PEGASIS is for each node to only communicate with their close neighbors and take turns being the leader for transmission to the sink. In PEGASIS, the locations of nodes are random, and each sensor node has the ability of data detection, wireless communication, data fusion and positioning. Energy load is distributed evenly among the sensor nodes in the network.

In PEGASIS, the nodes are organized to form a chain, which can either be concentratedly assigned by the sink and broadcast to all nodes or accomplished by the nodes themselves using a greedy algorithm. If the chain is formed by the nodes themselves, they can first get the location data of all nodes and locally compute the chain using the same greedy algorithm. During the process of chain formation in PEGASIS, it is assumed that all nodes have global knowledge of the network and the greedy algorithm is employed. The chain construction is commenced from the furthest node from the sink and the closest neighbor to this node will be the next node on the chain. When a node on the chain dies, the chain will be reconstructed in the same manner to bypass the dead node.

**Figure 8.**
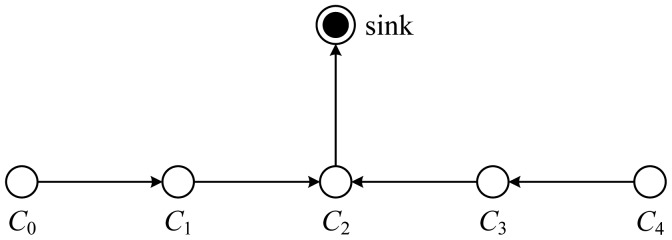
The Token Passing Scheme in PEGASIS.

For gathering data from sensor nodes in each round, each node receives data from one neighbor, fuses the data with its own, and transmits to the other neighbor on the chain. By moving from node to node, the fused data eventually are sent to the sink by the leader at a random position on the chain. The leader is important for nodes to die at random locations, in respect that the idea of nodes dying at random places is to enhance the robustness of the network. Alternatively, in each round, a control token passing approach initiated by the leader is used to start the data transmission from the ends of the chain. The scheme of data transmission in PEGASIS is shown in Figure 8. In this figure, if node *C*_2_ is the leader, it will pass the token along the chain to node *C*_0_ at first. Then, node *C*_0_ will pass its data toward node *C*_2_. After node *C*_2_ receives data from node *C*_1_, it will pass the token to node *C*_4_, and node *C*_4_ will pass its data towards node *C*_2_ with data fusion taking place along the chain.

According to above discussion, the following is the advantages of PEGASIS: (1) This protocol is able to outperform LEACH for different network sizes and topologies, because it reduces the overhead of dynamic cluster formation in LEACH, and decreases the number of data transmission volume through the chain of data aggregation; (2) The energy load is dispersed uniformly in the network. To ensure that the fixed sensor node is not select as the leader and thus to prevent the subsequent early death of this sensor node, all sensor nodes act as the leader in turn [70].

However, there are some disadvantages in PEGASIS: (1) It is the necessity of having a complete view of the network topology at each node for chain construction and that all nodes must be able to transmit directly to the sink. Thus, this scheme is unsuitable for those networks with a time varying topology [71]; (2) It is assumed that each sensor node can be able to communicate with the sink directly, but nodes usually use multi-hop communications with the sink in practical cases. Furthermore, long-range communications directly from the node to the sink can breed too much energy consumption; (3) The communication manner suffers from excessive delays caused by the single chain for distant nodes and a high probability for any node to become a bottleneck; (4) It is a difficult task for all nodes to maintain a complete database about the location of all other nodes in the network, furthermore the network is not very scalable because all nodes must have global knowledge of the network and employ the greedy algorithm.

#### 5.2.2. TEEN

Threshold sensitive Energy Efficient sensor Network protocol (TEEN) [27], proposed by Anjeshwar and Agrawal, is a hierarchical protocol whose main goal is to cope with sudden changes in the sensed attributes such as temperature. The protocol combines the hierarchical technique in line with a data-centric approach. The nodes sense their environment continuously, but the energy consumption in this algorithm can potentially be much less than that in the proactive network, because data transmission is done less frequently.

**Figure 9.**
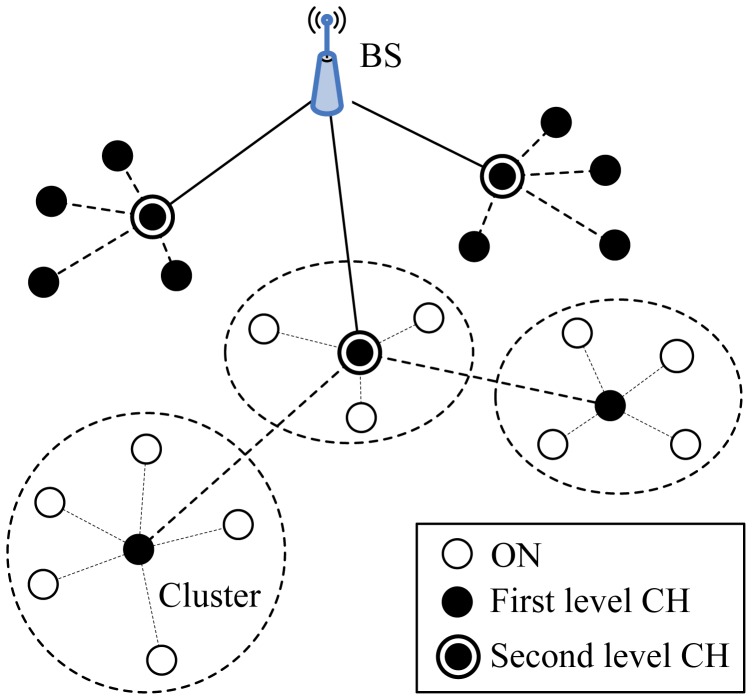
Illumination of the 2-tier Clustering Topology in TEEN.

In TEEN, a 2-tier clustering topology is built as illustrated in Figure 9 and two thresholds, hard threshold and soft threshold, are defined. The former threshold is a threshold value for the sensed attribute. It is the absolute value of the attribute beyond which, the node sensing this value must switch on its transmitter and report to its CH. The latter threshold is a small change in the value of the sensed attribute which triggers the node to switch on its transmitter and transmit.

In TEEN, a CH sends its members a hard threshold and a soft threshold. Thus the hard threshold tries to reduce data communications by allowing the nodes to transmit only when the sensed attribute is in the range of interest. The soft threshold further reduces data communications might have otherwise occurred when there is little or no change in the sensed attribute. At the expense of increased energy consumption, a smaller value of the soft threshold generates more accurate information of the network, thus users can control the trade-off between energy efficiency and data accuracy by the parameters adjustment. Moreover, the soft threshold can be varied and the users can change the fresh parameters as required at every cluster change time.

According to above discussion, TEEN has the following advantages: (1) Based on the two thresholds, data transmission can be controlled commendably, *i.e.*, only the sensitive data we demand can be transmitted, so that it reduces the energy transmission consumption and improves the effectiveness and usefulness of the receiving data; (2) TEEN is complement for reacting to large changes in the sensed attributes, which is suitable for reactive scenes and time critical applications.

However, there exist a few drawbacks in TEEN as follows: (1) It is not suitable for periodic reports applications since the user may not get any data at all if the values of the attributes may not reach the threshold [72]; (2) There exist wasted time-slots and a possibility that the BS may not be able to distinguish dead nodes from alive ones, because only when the data arrive at the hard threshold and has a variant higher than the soft threshold did the sensors report the data to the BS; (3) If CHs are not in the communication range of each other the data may be lost, because information propagation is accomplished only by CHs [73].

#### 5.2.3. APTEEN

The Adaptive Threshold sensitive Energy Efficient sensor Network protocol (APTEEN) [28], introduced by Manjeshwar and Agrawal, is an extension to TEEN and aims at both transmitting periodic data and reacting to time critical events. APTEEN, on the other hand, is a hybrid protocol that changes the periodicity or threshold values used in TEEN according to the requirement of users and the type of the application. APTEEN is based on a query system which allows three types of queries: historical, on-time, and persistent which can be used in a hybrid network. Moreover, QoS requirements are introduced for the on-time queries and minimum delay is achieved by a TDMA schedule with a special time slot assignment manner.

In APTEEN, CHs broadcast the following four parameters: (1) Attributes (A)—this is a set of physical parameters which the user is interested in obtaining data about; (2) Thresholds—this parameter consists of the hard threshold (HT) and soft threshold (ST). HT is a particular value of an attribute beyond which a node can be triggered to transmit data. ST is a small change in the value of an attribute which can trigger a node to transmit data again; (3) Schedule—this is a TDMA schedule, assigning a slot to each node; (4) Count time (CT)—it is the maximum time period between two successive reports sent by a node. It can be a multiple of the TDMA schedule length and it accounts for the proactive component.

The distinctive feature of APTEEN is to switch between proactive and reactive modes to transmit data. All nodes sense the environment continuously, but only those nodes which sense a data value at or beyond the hard threshold permit transmitting. Once a node senses a value at or beyond the hard threshold, it transmits data. If a node does not send data for a time period equal to the count time, it must sense and transmit the data again. In APTEEN, each CH aggregates the data from the MNs within its cluster and transmits the aggregated data to the BS. During the process of data aggregation, it is assumed that the data received from the corresponding MNs are sufficiently correlated, thus it reduces a large amount of redundancy of the data to be transmitted to the BS. Moreover, a modified TDMA schedule is used to implement the hybrid network by assigning each node in the cluster a transmission slot. Additionally, APTEEN offers a lot of flexibility by allowing the user to set the CT interval and the threshold values for energy consumption can be controlled by changing the CT as well as the threshold values.

The characters and advantages of APTEEN include: (1) APTEEN combines both proactive policies, which is alike that of LEACH, and reactive policies, which is alike that of TEEN. Accordingly it is suitable in both proactive and reactive applications; (2) It embodies a lot of flexibility by setting the count-time interval, and the threshold values for the energy consumption can be adjusted by changing the count time as well as the threshold values.

The main disadvantages of APTEEN are as follows: (1) There exist additional complexity required to implement the threshold functions and the count time; (2) Actually, both TEEN and APTEEN share the same drawbacks of additional overhead and complexity of cluster construction in multiple levels, implementing threshold-based functions, and dealing with attribute-based naming of queries—APTEEN more than TEEN [74].

#### 5.2.4. TTDD

The Two-Tier Data Dissemination (TTDD) approach, presented by Luo *et al.* [29], is a low-power protocol for efficient data delivery from multiple sources to multiple mobile sinks. It exploits a geographic routing based on grid of cells as the routing method. Instead of passively waiting for queries from sinks, sensor nodes can proactively establish a structure to set up forwarding information. Ultimately, the sensing field is figured as a set of grid points.

**Figure 10.**
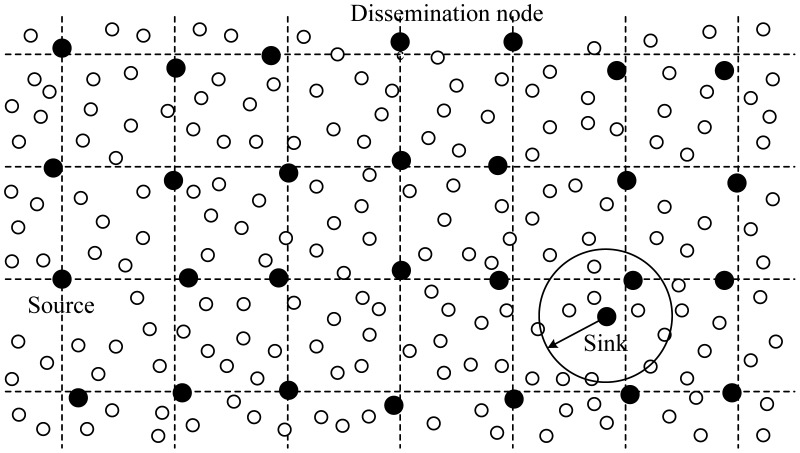
The Grid-based Topology in TTDD.

In TTDD, a source divides the field into a grid of cells and each cell is square. A source, at one crossing point of the grid, propagates data announcements to reach all the other crossings, called dissemination points, on the grid as shown in Figure 10. A source calculates the locations of its four neighboring dissemination points and sends a data announcement message to the four neighboring dissemination points using simple greedy geographical forwarding, *i.e.*, it forwards the message to the neighbor node that has the smallest distance to the neighboring dissemination point. Similarly, the neighbor node continues forwarding the data announcement message till the message stops at a node that is closer to the dissemination point than all its neighbors. During this process, each intermediate node stores the source information and this process continues until the message stops at the border of the network. After this process, the grid structure is obtained.

The sink can flood a query within a local area to discover nearby dissemination nodes. Once the query reaches a local dissemination node, it is forwarded on the grid to the upstream dissemination node from which this intermediate node receives data announcements. The query is forwarded by the upstream toward the source, until finally arrives at the source. During the above process, each dissemination node stores the location of the downstream dissemination node, thus this information is used to direct data back to the sink.

When a sink moves in the network, trajectory forwarding is employed to relay data to the mobile sink from its immediate dissemination node. In trajectory forwarding, each sink is associated with two sensor nodes: a primary agent and an immediate agent. A sink picks a neighboring sensor node as its primary agent which receives data directly from the immediate dissemination node, and subsequently relays data to the sink. Initially, the primary agent and the immediate agent are the same sensor node. When a sink is about to move out of the range of its immediate agent, it selects another neighboring node as its new immediate agent and sends the information of the new immediate agent to its primary agent, thus future data are forwarded to the new immediate agent.

The character and advantage of TTDD can be concluded as follows: (1) It deals with the problems caused by multiple mobile sinks and sink moving in large-scale WSNs; (2) Despite that it is effective in high mobility scenarios, the overhead to build and maintain the overlay is significant, especially in periodic reporting scenarios, which are more traffic intensive than event-based reporting. Therefore, TTDD is better suited to event-detecting WSNs with sporadic rather than continuous traffic [75].

There are some disadvantages in TTDD as follows: (1) The routing of a forwarding path in TTDD is not the shortest path, thus it may lead to large latency for the long path; (2) The grid structure formation and query flooding cost large energy consumption; (3) TTDD requires that sensor nodes are stationary and location-aware and assumes the availability of an accurate positioning system that may not yet available in a real WSN. If mobile sensor nodes are allowed to move in the network, how would TTDD perform is still an open question.

#### 5.2.5. CCS

The Concentric Clustering Scheme (CCS) has been proposed in [30] by Jung *et al.* to reduce the energy consumption loopholes in PEGASIS. The main idea of CCS is to consider the location of the BS to enhance its performance and to prolong the lifetime of the network.

**Figure 11.**
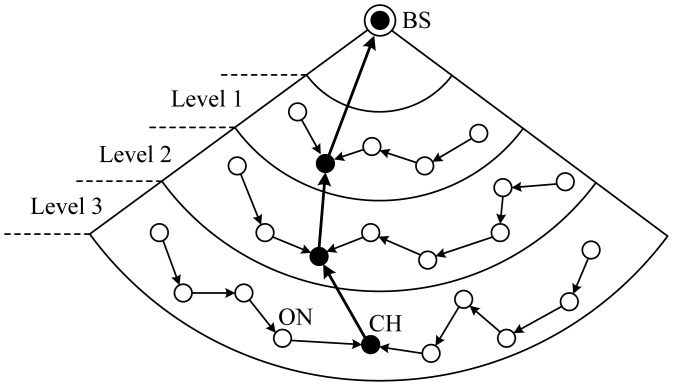
Illumination of the Data Transmission Scheme in CCS.

In CCS, the network is divided into a variety of concentric circular tracks which represent different clusters and each circular track is assigned with a level. The track nearest to the BS is assigned with level-1 and the level number increases with the increase of the distance to the BS. Thus, each node in the network is assigned with its own level. Besides, chains are constructed within the track as that in PEGASIS. One of the nodes on the chain at each level area is selected as a CH. A CH in level *L* is selected with node number obtained by calculating *i* mod *M_L_*, where *M_L_* represents the number of nodes that have the same level in *i* round. Data transmission in CCS is based on the process of PEGASIS protocol. After CH selection, each CH transmits the data of its own location to both the upper and lower level CH in one grade. In the process of the data transmission, all nodes in each level transmit the data to the nearest node from themselves along the chain. The node receives the data and fuses its own data and transmits these data to the next node. Therefore, the CH receives at most two data messages. Subsequently, the CH in each level transmits the data to the lower CH. At last, level 1 CH transmits these data to the BS. The data transmission scheme in CCS is shown in Figure 11.

Compared to PEGASIS, CCS embodies the advantaged as follows: (1) The distance over which the data can be transmitted to the BS from the CH is reduced in CCS. Hence, a considerable amount of energy is saved on account of the reduction of transmission distance in CCS [76]; (2) The network is divided into a series of concentric clusters, and the reverse data flow from the BS is also reduced. Thus, a considerable amount of energy is also conserved during data transmission.

However, there are some disadvantages to be considered as follows: (1) Node distribution in each level is unbalanced, thus the levels with small number of nodes will deplete their energy first, in that the probability of election to be a CH is high; (2) Residual energy is not considered for CH election, which may lead to unbalanced energy consumption among all nodes; (3) Chain-based protocols, such as PEGASIS and CCS, enable nodes to communicate with their closest neighbor by using low radio power, but the long chain would cause large delay [77]; (4) The CH selection for next hop is based on the location rather than the residual energy of nodes, thus energy of CH may dissipates quickly on the path among CHs, and even energy hole will appear in the network.

#### 5.2.6. HGMR

Hierarchical Geographic Multicast Routing (HGMR), proposed in [31] by Koutsonikolas *et al.*, is a location-based multicast protocol. This protocol seamlessly incorporates the key design concepts of the Geographic Multicast Routing (GMR) [78] and Hierarchical Rendezvous Point Multicast (HRPM) protocols [79], and optimizes them by providing forwarding energy efficiency as well as scalability to large-scale WSNs.

HGMR starts with a hierarchical decomposition of a multicast group into subgroup of manageable size by means of the key concept of mobile geographic hashing of HRPM. Within each subgroup, HGMR adopts the local multicast scheme of GMR to forward data packets along multiple branches of the multicast tree in one transmission. In HGMR, the multicast group is divided into subgroups using the mobile geographic hashing idea: the deployment area is recursively partitioned into a number of *d*^2^ equal-sized square sub-domains called cells, where *d* is decomposition index depending on the encoding overhead constraints, and each cell comprises a manageably-sized subgroup of members. In each cell there is an Access Point (AP) responsible for all members in that cell, and all APs are managed by a Rendezvous Point (RP).

**Figure 12.**
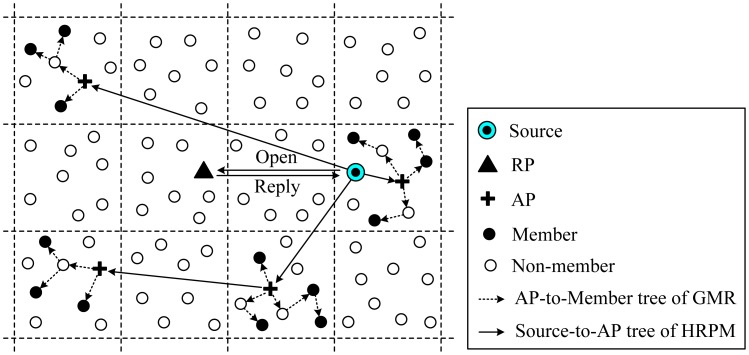
Illumination of data delivery in HGMR.

In order to join a hierarchically decomposed multicast group, a node generates the hashed location for the RP and sends a join message to that location. After receiving the value of decomposition index *d* from the RP, the node invokes the hash function with *d* and its location, to achieve the hashed location of the AP of the cell it belongs to. Consequently, the source builds an overlay tree, the Source-to-AP tree, whose vertices are active APs in a topology graph, and another overlay tree, the AP-to-Member tree is also built from the AP, considering each member as the vertex. When a source needs to transmit data packets, it utilizes the unicast-based forwarding strategy of HRPM to propagate data packets to each AP along the Source-to-AP overlay tree. In each cell, instead of constructing an AP-to-Member overlay tree, HGMR uses the cost over progress optimizing broadcast algorithm of GMR to select the next relay nodes at each hop. By adjusting the value for the decomposition index *d*, the number of members an AP is responsible for does not increase too much. Hence, the use of GMR within each cell instead of the unicast-based forwarding strategy of HRPM contributes to reduce the number of transmissions. When routing to a hashed location (RP or AP), HGMR uses the face routing of HRPM, while when routing from an AP to a set of group members within a cell, it uses the multicast face routing of HRPM. The data delivery in HGMR is shown in Figure 12.

The main merits of HGMR can be summarized as follows: (1) The membership management of HGMR is very simple and easy without additional cost due to the geographic hashing algorithm; (2) According to the number of the nodes which play the different roles, the data transmission methods for different hierarchies in HGMR make the routing energy-efficient in a way; (3) HGMR is free of the scalability problem in that only manageable destinations exist in a cell.

However, there are a few drawbacks in HGMR as follows: (1) The simple network partition into a set of cells may lead to sub-optimal routing paths from the root node to multicast group members; (2) All transmissions are concentrated to APs. APs can be changed to another node by hash function, but it is too limited in a cell, and may bring on unbalanced energy consumption around APs; (3) HGMR makes the routing paths inefficient to some extent, in that data packets are forwarded from the upper APs to the lower APs hierarchically, whether the lower APs are closer to the source than the upper APs or not [80].

## 6. Comparison of Different Clustering Routing Protocols in WSNs

In this section, we compare the different clustering routing algorithms for WSNs. We summarize the categories and differences of the clustering routing protocols in WSNs according to a variety of clustering attributes as shown in Table 2. Furthermore, we compare the different clustering routing approaches in WSNs based on a few important metrics in Table 3.

## 7. Summary and Conclusions

Wireless sensor networks (WSNs) have attracted significant attention over the past few years, and can be employed in a wide spectrum of applications in both civilian and military scenarios. The design of effective, robust, and scalable routing protocols for WSNs is a challenging task. On the other hand, clustering routing algorithms, generally, can well match the constraints and the challenges of WSNs. As a result, it is clearly seen so far that, significant efforts have been made in addressing the techniques to design effective and efficient clustering routing protocols for WSNs in the past few years.

In this paper, we have presented a rather extensive survey on clustering routing protocols in WSNs. We have also developed a novel taxonomy of clustering routing methods for WSNs based on rather detailed clustering attributes. Finally, we have systematically analyzed a few classical WSN clustering routing protocols in deep, and compared these different approaches based on our taxonomy and some primary metrics.

To conclude, we want to sketch some future directions for the field. Firstly, further research would be needed to address QoS problem of clustering routing, which mainly exists in real-time applications, such as battle-target tracking, emergent-event monitoring, and *etc.* Recently there is very little research focuses on handling QoS requirements in the resource-constrained WSN environment. Moreover, further studies are necessary to settle the problem of node mobility, including the sink and sensor nodes. There are a lot of node-mobility applications, such as battle scenarios, thus how to handle the overhead of node mobility and topology changes must be further taken into account. Finally, with the increase of the network scale in WSNs, more redundant information is created and a certain degree of redundancy may be desirable for increasing reliability of the network. Thus, a trade-off between redundancy reduction and redundancy utilization is still an open question.

We hope that this will encourage protocol researchers and designers to take into account the various characteristics of the clustering routing methods when designing an effective clustering routing protocol, and that more work would be done in the future directions for the field. We believe that we will witness a great diffusion of clustering routing solutions for more comprehensive applications for WSNs.

## Figures and Tables

**Table 1.** Summary of Previous Surveys on Clustering Routing Protocols in WSNs

**Year**	**Authors**	**Literature**	**Main contributions**
2006	Arboleda. *et al.*	Comparison of clustering algorithms and protocols for wireless sensor networks [33]	➀ Discussion of basic concepts related to the clustering process ➁ Analysis of LEACH-based protocols ➂ Survey of proactive and reactive algorithms in WSNs
2007	Abbasi *et al.*	A survey on clustering algorithms for wireless sensor networks [32]	➀ Presentation of a taxonomy of typical clustering schemes ➁ Survey of variable convergence time clustering protocols and constant convergence time clustering algorithms in WSNs ➂ Comparison of popular clustering methods
2008	Kumarawadu *et al.*	Algorithms for node clustering in wireless sensor networks: a survey [34]	➀ Presentation of a classification based on cluster-formation parameters and CH election criteria ➁ Discussion of the key design challenges of WSN clustering ➂ Analysis of the performance issues related WSN clustering
2008	Deosarkar *et al.*	Cluster head selection in clustering algorithms for wireless sensor networks: a survey [35]	➀ Discussion of CH selection strategies based on the classification of deterministic scheme, adaptive scheme and combined metric scheme ➁ Comparison of the costs of CH selection with respect to cluster formation, distribution of CHs and creation of clusters
2009	Jiang *et al.*	Towards clustering algorithms in wireless sensor networks-a survey [36]	➀ Presentation of a classification of WSN clustering schemes based on 8 clustering attributes ➁ Analysis of popular WSN clustering algorithms ➂ Comparison of popular WSN clustering algorithms
2010	Maimour *et al.*	Cluster-based routing protocols for energy-efficiency in wireless sensor networks [37]	➀ Presentation of a classification of WSN clustering algorithms ➁ Discussion of typical WSN clustering protocols based on pre-established and on-demand manners ➂ Summary of some future research directions
2010	Lotf *et al.*	Hierarchical routing in wireless sensor networks: a survey [38]	➀ Discussion of typical clustering routing protocols for WSNs ➁ Comparison of clustering routing algorithms based on energy consumption and network lifetime
2010	Boyinbode *et al.*	A survey on clustering algorithms for wireless sensor networks [39]	➀ Discussion of the main challenges for WSN clustering ➁ Analysis of popular clustering algorithms for WSNs ➂ Comparison of popular WSN clustering algorithms based on many attributes
2011	Wei *et al.*	Cluster-based routing protocols in wireless sensor networks: a survey [40]	➀ Presentation of a classification based on three attributes ➁ Discussion of the challenges in WSN clustering ➂ Analysis of popular clustering routing techniques
2011	Xu *et al.*	Comparison study to hierarchical routing protocols in wireless sensor networks [41]	➀ Analysis of popular clustering routing protocols in WSNs ➁ Comparison of popular WSN clustering routing algorithms
2011	Joshi *et al.*	A survey of hierarchical routing protocols in wireless sensor network [42]	➀ Discussion of familiar clustering routing algorithms in WSNs ➁ Comparison of familiar WSN clustering routing protocols based on energy conservation and network lifetime
2012	Haneef *et al.*	Design challenges and comparative analysis of cluster based routing protocols used in wireless sensor networks for improving network life time [43]	➀ Discussion of the challenging factors in WSN clustering ➁ Presentation of a classification of routing protocols in WSNs ➂ Analysis of classical WSN clustering routing protocols ➃ Comparison of classical clustering routing algorithms for WSNs

**Table 2.** Classification of Different Clustering Routing Protocols in WSNs.

**Clustering Routing Protocols**		**LEACH**	**HEED**	**DWEHC**	**PANEL**	**TL-LEACH**	**UCS**	**EECS**	**EEUC**
Cluster characteristics	Variability of cluster count	variable	variable	variable	fixed	variable	variable	variable	variable
	Uniformity of cluster sizes	even	even	even	even	even	uneven	uneven	uneven
	Intra-cluster routing	single-hop	single-hop	multiple-hop	single-hop	single-hop	single-hop	single-hop	single-hop
	Inter-cluster routing	single-hop	single-hop multiple-hop	single-hop	multiple-hop	multiple-hop	multiple-hop	single-hop	multiple-hop
Cluster-head characteristics	Existence	cluster-head based	cluster-head based	cluster-head based	cluster-head based	cluster-head based	cluster-head based	cluster-head based	cluster-head based
	Difference of capabilities	homogeneous	homogeneous	homogeneous	homogeneous	homogeneous	heterogeneous	homogeneous	homogeneous
	Mobility	stationary	stationary	stationary	stationary	stationary	stationary	stationary	stationary
	Role	relay aggregation	relay aggregation	relay aggregation	relay aggregation	relay aggregation	relay aggregation	relay aggregation	relay aggregation
Clustering process	Control manners	distributed	distributed	distributed	distributed	distributed	distributed	distributed	distributed
	Execution nature	probabilistic	iterative	iterative	probabilistic	probabilistic	probabilistic	probabilistic	probabilistic
	Convergence time	constant	constant	constant	constant	constant	constant	constant	constant
	Parameters for CH election	adaptive	adaptive	adaptive	adaptive	adaptive	adaptive	adaptive	adaptive
	Proactivity	proactive	proactive	proactive	proactive	proactive	proactive	proactive	proactive
	Objectives	load balancing	load balancing	load balancing	load balancing reliability	load balancing lifetime extension	load balancing lifetime extension	load balancing periodical data communications	load balancing
Entire proceeding of the algorithm	Algorithm stages	cluster construction	cluster construction	cluster construction	cluster construction	cluster construction	cluster construction	cluster construction	cluster construction
Cluster characteristics	Variability of cluster count	variable	variable	variable	fixed	variable	variable	variable	variable
	Uniformity of cluster sizes	even	even	even	even	even	even	uneven	even
	Intra-cluster routing	single-hop	single-hop	multiple-hop	simple-hop	single-hop	single-hop	multiple-hop	single-hop
	Inter-cluster routing	single-hop	single-hop multiple-hop	single-hop	multiple-hop	multiple-hop	multiple-hop	multiple-hop	multiple-hop
Cluster-head characteristics	Existence	cluster-head based	cluster-head based	no-cluster-head based	cluster-head based	cluster-head based	cluster-head based	cluster-head based	cluster-head based
	Difference of capabilities	homogeneous	homogeneous	N/A	homogeneous	homogeneous	heterogeneous	homogeneous	homogeneous
	Mobility	stationary	stationary	N/A	stationary	stationary	stationary	stationary	stationary
	Role	relay aggregation	relay aggregation	N/A	relay aggregation	relay aggregation	relay aggregation	relay aggregation	relay aggregation
Clustering process	Control manners	distributed	centralized	distributed	distributed	distributed	distributed	distributed	distributed
	Execution nature	iterative	iterative	probabilistic	probabilistic	probabilistic	probabilistic	probabilistic	probabilistic
	Convergence time	constant	constant	constant	constant	constant	constant	constant	constant
	Parameters for CH election	adaptive	adaptive	adaptive	adaptive	adaptive	adaptive	adaptive	adaptive
	Proactivity	proactive	proactive	proactive	reactive	proactive reactive	proactive	proactive	proactive
	Objectives	load balancing	load balancing	load balancing	reactive scenes lifetime extension	proactive scenes reactive scenes	scenes of multiple mobile sinks	lifetime extension	lifetime extension
Entire proceeding of the algorithm	Algorithm stages	cluster construction	cluster construction	data transmission	data transmission	data transmission	data transmission	data transmission	data transmission

**Table 3.** Comparison of Different Clustering Routing Protocols in WSNs.

**Ptotocol Name**	**Energy Efficiency**	**Cluster Stability**	**Scalability**	**Delivery Delay**	**Load Balancing**	**Algorithm Complexity**
LEACH	very low	moderate	very low	very small	moderate	low
HEED	moderate	high	moderate	moderate	moderate	moderate
DWEHC	very high	high	moderate	moderate	very good	moderate
PANEL	moderate	low	low	moderate	good	high
TL-LEACH	low	moderate	moderate	small	bad	low
UCS	very low	high	low	small	bad	moderate
EECS	moderate	high	low	small	moderate	very high
EEUC	high	high	high	moderate	good	high
ACE	moderate	very low	moderate	small	moderate	very high
BCDCP	very low	high	very low	small	good	very high
PEGASIS	low	low	very low	very large	moderate	high
TEEN	very high	high	low	small	good	high
APTEEN	moderate	very low	low	small	moderate	very high
TTDD	very low	very high	low	very large	good	low
CCS	low	low	low	large	very bad	moderate
HGMR	low	high	very high	moderate	bad	low
